# Amniotic fluid embolism: lessons for rapid recognition and intervention

**DOI:** 10.4322/acr.2021.311

**Published:** 2021-08-20

**Authors:** Larry Nichols, Rema Elmostafa, Angela Nguyen, Keisha R. Callins

**Affiliations:** 1 Mercer University School of Medicine, Department of Pathology and Clinical Science Education, Macon, GA, USA

**Keywords:** Amniotic fluid embolism, Maternal mortality, Autopsy, Extracorporeal membrane oxygenation

## Abstract

Amniotic fluid embolism is a rare, often fatal complication of labor and delivery. The classic presentation is the sudden onset of a triad of clinical manifestations: hypoxia, hypotension and coagulopathy. Understanding of the syndrome as an immunologically mediated, complicated and often catastrophic maternal response to fetal or placental antigens is coming into focus. New treatments such as extracorporeal membrane oxygenation (ECMO) and better use of old treatments such as transfusion offer hope, but the condition is often rapidly fatal, so saving the maternal and fetal lives depends on rapid recognition of the syndrome. This series of three cases illustrates the clinical features enabling the rapid recognition needed for successful treatment of amniotic fluid embolism syndrome.

## INTRODUCTION

Labor and delivery have the potential to become a critical experience for mother and baby. The obstetrician must always be on high alert for the rare emergency. Sudden cardiovascular collapse during labor and delivery can be due to pulmonary thromboembolism, hemorrhage, venous air embolism, anaphylaxis, high cephalad spread of epidural anesthesia, peripartum cardiomyopathy, eclampsia or amniotic fluid embolism.^1^ Among these complications, amniotic fluid embolism remains one of the most feared because of its suddenness, severity and frequently fatal outcome. Emerging life-saving treatments for amniotic fluid embolism make it more promising to successfully treat this life-threatening cause of cardiovascular collapse during labor and delivery. This series of three cases is reported in detail to highlight the variability in presentation and provide a didactic review to encourage rapid recognition and intervention.

## CASE 1

This 35-year-old mother of two was admitted to an urban hospital in the Midwest USA at 37 4/7 weeks gestation at 09:21 with spontaneous rupture of membranes since 04:00. She was having irregular mild contractions. Group B Streptococcus had been isolated. Her outpatient medications included only salmeterol/fluticasone inhaler for asthma, and prenatal vitamins. The patient's temperature was 36.3^o^ C, pulse 86/minute, blood pressure 135/85 mm Hg and respirations 20/minute. Fetal heart rate was normal at 120-130/minute. At 10:15 the patient's hemoglobin was 11.6 g/dL. (RR: 11.7-15.7 g/dL). An intravenous infusion of lactated Ringer's solution was started, and the patient received prophylactic penicillin. Oxytocin augmentation was started. A total of 24 MU of oxytocin was given from 13:30 to 18:15. Labor proceeded uneventfully, with the cervix dilated to 5 cm, until about nine hours after admission.

At approximately 18:15, according to the husband, the patient sat up and reported the sudden onset of deep back, rib and flank pain, and shortness of breath. The husband ran out of the room to summon help. An anesthesiologist, who had been in the next room, entered the room and found the patient unresponsive and cyanotic, with tonic-clonic seizure activity. The patient initially had a palpable pulse and sustained respiratory effort. She was given 100% oxygen by facemask. Fetal heart tones were difficult to auscultate externally and a fetal scalp electrode was applied. The fetal heart rate was difficult to ascertain with the electrode. An emergent sonogram showed a heart rate around 60/minute and dropping. The patient became apneic and cyanotic. At approximately 18:30 she became pulseless. Cardiopulmonary resuscitation was initiated. The patient was intubated and ventilated with 100% oxygen, but remained cyanotic. An emergency cesarean section was performed. At 18:35 the baby was delivered. At 18:36 the placenta was delivered. The baby's Apgar scores were 4 at 1 minute and 8 at 5 minutes.

The patient's cardiovascular status did not improve after delivery. She remained without a carotid pulse and unresponsive to atropine and epinephrine. The uterus remained atonic. Endotracheal epinephrine briefly boosted the heart rate to 130/minute, but still without a pulse. A right femoral line was placed. Additional fluid infusion was to no avail. At 18:59 the patient developed ventricular fibrillation. Despite repeated defibrillation, at 19:02 she became asystolic. At 19:09 attempted resuscitation was halted and the patient pronounced dead, 54 minutes after the onset of dyspnea. Blood drawn while the patient was asystolic showed: hemoglobin 6.6 g/dL, platelets 26,000/mm^3^ (RR: 156,000-369,000/mm^3^), sodium 142 mEq/L (RR: 136-145 mEq/L), potassium 4.2 mEq/L (RR: 3.5-5.1 mEq/L), chloride 105 mEq/L (RR: 95-110 mEq/L), bicarbonate 17.1 mEq/L (RR: 21-31 mEq/L), magnesium 2.3 mg/dL (RR: 1.6-2.5 mg/dL), and white blood cell count 6,000/mm^3^ (RR: 3,800-10,600/mm^3^), with 51% lymphocytes, 38% neutrophils, 5% monocytes, 5% bands and 1+ toxic granulations.

### Case 1 Autopsy Findings

Autopsy revealed widespread congestion of the internal organs, scattered petechial hemorrhages in kidneys and colon, mild congestive hepatosplenomegaly and mild four-chamber cardiac dilatation. The lungs were markedly congested and exuded a small amount of frothy fluid on sectioning. The bronchi contained a moderate amount of frothy mucoid fluid. The left lung weighed 390 grams (RR: 325-480 grams) and right lung 430 grams (RR: 360-570 grams). Microscopic examination revealed small pulmonary blood vessels markedly distended with watery fluid, numerous neutrophils, and moderate numbers of monocytes, lymphocytes and platelets. There were rare squamous cells in small pulmonary blood vessels. There were rare platelet-fibrin thrombi in renal glomeruli. The placenta showed a 5 cm tear in the disc at the point of rupture and chronic villitis of unknown etiology involving about 30% of the villi along the decidual plate.

## CASE 2

This 34-year-old gravida 3 para 2 woman had a 9-year-old and a 7-year-old from uncomplicated pregnancies. On a prenatal visit 6 weeks prior, her hemoglobin was 12 g/dL and platelets 254,000/mm^3^. The patient was admitted to a rural community hospital in the Midwest USA in labor at 38 6/7 weeks gestation at 15:00, with temperature 36.8^o^ C, blood pressure 132/81 mm Hg and oxygen saturation 96%. She was given epidural anesthesia and started on low-dose oxytocin.

Shortly after midnight, at 00:20, the patient abruptly complained of dyspnea, blurred vision and a headache. She then developed chest pain. Her eyes rolled back and she became marginally responsive to verbal stimuli. The fetal heart rate became labile and varied from 90 to 150/minute. The patient’s oxygen saturation dropped to 89%. She was given supplemental oxygen. Epidural anesthesia and oxytocin were discontinued. Her saturation increased to 95% and her blood pressure was 120/84 mm Hg.

The patient’s oxygen saturation, heart rate and blood pressure became labile, and she became unresponsive. At 00:36, her saturation decreased to 74%. At 00:48, the fetal heart rate dropped to 60-70/minute. The baby was delivered with forceps at 01:00, with Apgar scores of 5 at one minute, 6 at 5 minutes and 9 at 10 minutes. The placenta was delivered at 01:08, apparently complete and intact.

Following delivery of the baby and placenta, at 01:16, the patient’s heart rate increased to 174/minute and blood pressure decreased to 57/37 mm Hg, with saturation 94%. She was given ephedrine. Her heart rate decreased to 53/minute, with blood pressure 58/37 mm Hg and saturation 78% at 01:24. She then became tachycardic and hypertensive, with heart rate 187/minute, blood pressure 145/109 mm Hg and saturation 99% at 01:35. Her saturation fell to 82%, with heart rate 170/minute and blood pressure 93/79 mm Hg at 01:40. Her fundus was boggy and bleeding. A second intravenous line was placed in right femoral vein and the patient was transferred to an intensive care unit (ICU).

On arrival in the ICU at 02:00, the patient’s saturation was 99%. Blood drawn at 02:10 showed hemoglobin 7.1 g/dL, white blood cell count 34,500/mm^3^ (38% neutrophils, 46% bands, 14% lymphocytes, 1% monocytes, 1% metamyelocytes), platelets 145,000/mm^3^, partial thromboplastin time (PTT) 87.3 seconds (RR: 25.3-35 seconds) and fibrinogen <15 mg/dL (RR: 267-484 mg/dL). The patient’s saturation was labile, soon dropping to 79% and then increasing to 99%. Her heart rate ranged from 133/minute to 187/minute. She had hypotension treated with infusions of dopamine, dobutamine and phenylephrine. At 03:15, the patient’s hemoglobin was 3.3 g/dL, platelets 95,000/mm^3^ and white blood cell count 29,200/mm^3^. She was transfused red blood cells, platelets, fresh frozen plasma and cryoprecipitate. The patient was intubated and mechanically ventilated. At 03:59, arterial blood showed pH 6.81, PCO2 17 mm Hg and PO2 432 mm Hg. At 05:17, glucose was 334 mg/dL (RR: 70-110 mg/dL), blood urea nitrogen 10 mg/dL (RR: 5-20 mg/dL), creatinine 1.4 mg/dL (RR: 0.5-1.4 mg/dL), sodium 142 mEq/L, potassium 5.4 mEq/L, chloride 110 mEq/L, bicarbonate 7 mEq/L, calcium 6.7 mg/dL (RR: 8.5-10.5 mg/dL), bilirubin 3 mg/dL (RR: 0.1-1.2 mg/dL), alkaline phosphatase 124 U/L (RR: 40-125 U/L), alanine aminotransferase 77 U/L (RR: <40 U/L), aspartate aminotransferase 125 U/L (RR: <40 U/L), and albumin 2.5 g/dL (RR: 3.5-5.5 g/dL).

The patient’s urine output dropped until, by 10:00, she was anuric. Line placement for dialysis was unsuccessful. The patient became hypoxemic; arterial blood, drawn at 10:05, on mechanical ventilation with 100% oxygen, showed pH 7.33, PCO2 22 mm Hg and PO2 36 mm Hg. The patient had severe bleeding from the uterus. The uterus was packed, but the bleeding overwhelmed the packing within 10 minutes. At 11:00, an emergency hysterectomy was performed for uncontrollable uterine bleeding. Vaginal tears were sutured. At 11:25, the patient’s hemoglobin was 13 g/dL, white blood cell count 14,200/mm^3^ (26% neutrophils, 51% bands, 16% lymphocytes, 4% monocytes, 3% metamyelocytes) and platelets 78,000/mm^3^.

Twenty minutes following surgery, the patient suffered a severe deterioration in clinical status. She began developing cardiac arrhythmias including bigeminy, junctional rhythms and ventricular tachycardia. The patient was eventually transfused 18 units of red blood cells, 12 units of platelets, 7 units of fresh frozen plasma and 24 units of cryoprecipitate. At 13:19, her hemoglobin was 14.8 g/dL, white blood cell count 16,600/mm^3^, platelets 88,000/mm^3^, international normalized ratio (INR) 2.1 (RR: 0.9-1.1), PTT 61.3 seconds and fibrinogen 150 mg/dL. At 13:24, arterial blood showed pH 7.09, PCO2 48 mm Hg and PO2 30 mm Hg, on 100% oxygen and 15 cm H_2_O of positive end-expiratory pressure. The patient’s clinical condition continued to deteriorate despite heroic efforts and she died at 13:55, 13 hours and 35 minutes after the onset of dyspnea.

### Case 2 Autopsy Findings

**Figure 1:**
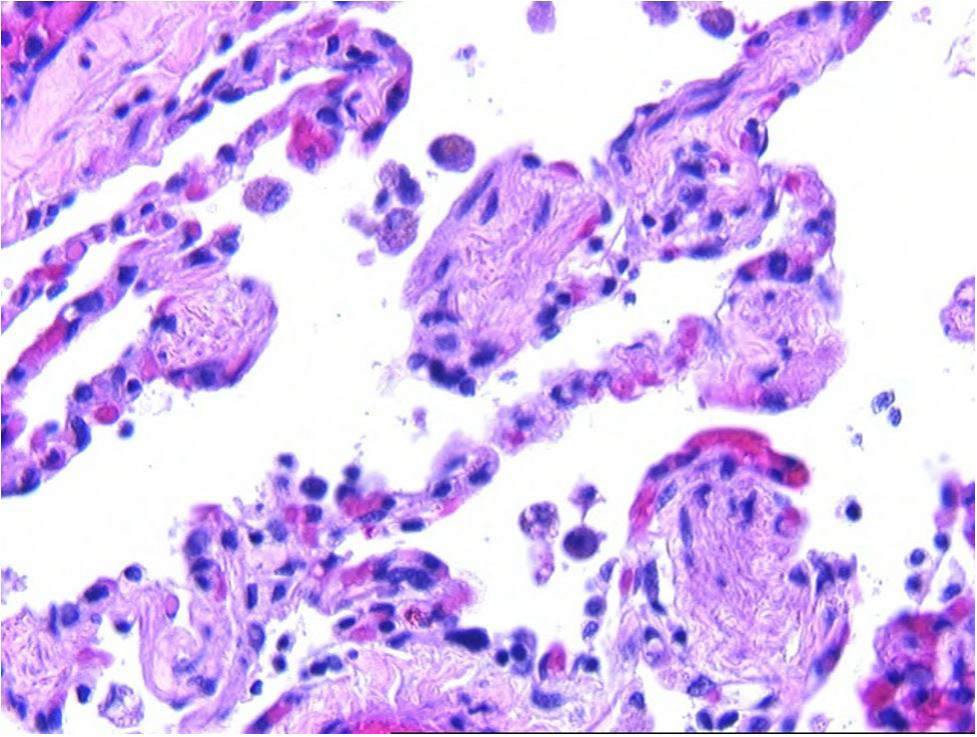
Microscopic examination revealed flat squamous cells in the lumens of small pulmonary blood vessels (H&E, 100X).

**Figure 2:**
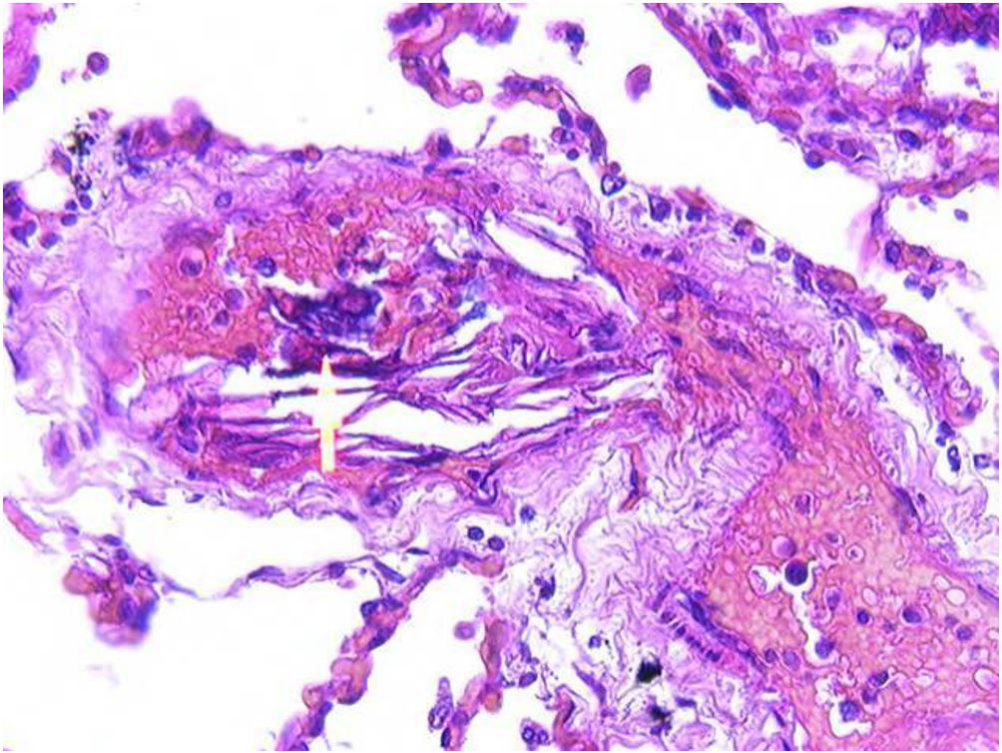
Microscopic examination revealed small calcifications (indicated by the arrow) in addition to flat squamous cells in the lumens of small pulmonary blood vessels (H&E, 100X).

**Figure 3:**
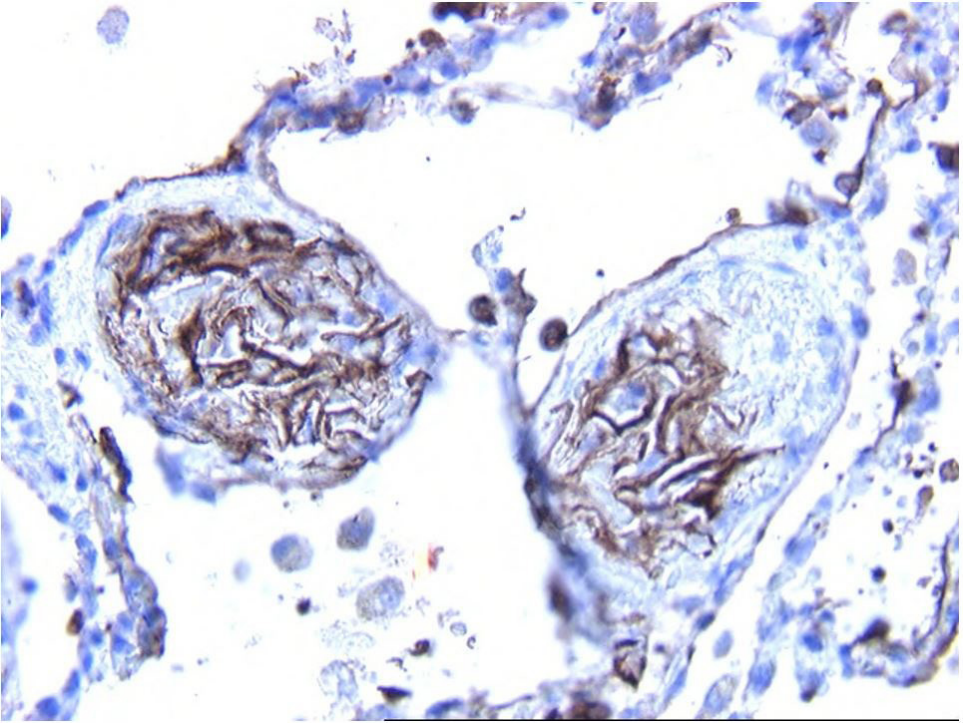
Immunohistochemical staining for cytokeratin AE3 confirmed the epithelial nature of the flattened squamous cells in the small pulmonary blood vessels (100X).

Autopsy revealed numerous small pulmonary blood vessels containing squamous cells (Figure 1), many surrounded by cleared space in the luminal blood, and some accompanied by small calcifications (Figure 2). The squamous cells were positive on immunohistochemical stain for cytokeratin AE3 (Figure 3). Mucinous material was identified in scattered small pulmonary blood vessels on mucicarmine stain. Autopsy revealed many sites of hemorrhage in heart, stomach, duodenum, kidneys, bladder, vagina and pelvis, and 1000 mL of partially clotted blood in the abdominal cavity. The pleural cavities contained serous fluid, 210 mL on the left and 550 mL on the right. There was pulmonary congestion and edema, with a combined lung weight of 1080 grams. There were occasional fibrin thrombi in renal glomeruli.

Examination of the placenta showed an intact complete 496-gram placenta with no significant abnormalities. Examination of the uterus from the hysterectomy showed decidualized endometrium and cervical foci of hemorrhage with blood vessels containing anucleate squames.

## CASE 3

This 43-year-old woman had a spontaneous vaginal delivery at term, 4 years prior, and an abortion at 6 weeks gestation, 14 years prior. She also had a history of gestational diabetes mellitus, a cholecystectomy (8 years prior) and mitral valve prolapse with mitral regurgitation. She had asthma, first diagnosed at age 5, but had not required medication since 10 months prior (when she had pneumonia). The patient conceived by in vitro fertilization. Transabdominal ultrasound 13.1 weeks after conception showed a 15.1week intrauterine gestation, with an echogenic intracardiac focus in the fetal left ventricle, and an anterior, low-lying placenta; amniocentesis revealed a normal male karyotype and an alpha fetoprotein level of 17.63 mcg/mL (0.98 MoM). The patient had gestational diabetes mellitus diagnosed at 33 weeks gestation.

At 36-4/7 weeks gestation, the patient presented at 23:10 to an urban hospital emergency department in the Midwest USA with an exacerbation of asthma. She had worsening dyspnea for approximately one week, associated with sharp back pain, a cough with deep breathing, and an episode of syncope with a fall. She had taken no medication for this until the day before, when she started using her inhalers. On examination, her temperature was 35.7^o^ C, heart rate 100/minute, blood pressure 134/83 mm Hg, respirations 20/minute and saturation 93% on room air. She had shallow respirations, but no wheezing. Her saturation was 96% on supplemental oxygen at 1 L/minute and 98% on 5 L/minute by nasal cannula. She was given a combination albuterol and ipratropium treatment. She was discharged at 02:45 with combination fluticasone and salmeterol inhaler and albuterol inhaler. Over the course of the day, her dyspnea worsened.

At 36-6/7 weeks gestation, she returned to the emergency department at 00:40, about 22 hours after her discharge. Her temperature was 35.4^o^ C, pulse 115/minute, blood pressure 112/84 mm Hg, respiratory rate 24/minute and oxygen saturation 94% on room air. She had diffuse pulmonary crackles and mild extremity edema. Her oxygen saturation increased to 96% on supplemental oxygen at 5 L/minute by nasal cannula. At 01:25 she received nebulizer treatment with no improvement. The respiratory therapist heard pulmonary rales over the right middle and lower lobes, and little air movement. A fetal heart monitor was applied. At 02:00 her white blood cell count was 9,600/mm^3^ (neutrophils 75%, lymphocytes 19%, monocytes 5%), hemoglobin 13.9 g/dL, and platelets 160,000/mm^3^. Chest x-ray revealed a right lower lobe infiltrate. Fetal heart tracings showed a reactive pattern at a rate of 120-160/minute. At 02:50 the patient was comfortable and able to speak in complete sentences without dyspnea. At 04:00 she was transferred to an inpatient hospital unit.

At 04:30 the patient's temperature was 35.8^o^ C, heart rate 105/minute, blood pressure 104/65 mm Hg, respirations 24/minute and saturation 97% on oxygen at 4 L/minute. She reported dyspnea and back pain she rated 10/10. She had decreased urine output. At 04:45 the patient complained of nausea. Her saturation was 98% on oxygen at 4 L/minute. A bladder catheter was placed. At 05:25 she received another nebulizer treatment with no improvement. She complained of dizziness. At 05:30 she complained of not feeling well, light-headedness and sweatiness. Her blood pressure was 79/62 mm Hg, with unchanged heart rate and respiratory rate. Her glucose was 137 mg/dl. She was given intravenous normal saline for her hypotension, a 500 ml bolus followed by 125 ml/hour. At 06:00 the patient was given azithromycin (500 mg intravenously) for suspected community-acquired pneumonia and she had dyspnea, nausea and a 50 ml emesis. Her temperature was 35^o^ C, pulse 95/minute, blood pressure 90/50 mm Hg, respiratory rate 18/minute and saturation 98% on oxygen at 4 L/minute. She was anxious and diaphoretic. She had pulmonary crackles. She was given ondansetron. At 06:30 her pulse was 110/minute, blood pressure 87/62 mm Hg and respirations 30/minute. At 07:30 the patient was upset, complaining of back pain and discomfort from having a bladder catheter. She said the antibiotic "made me feel terrible; get me out of here." She was refusing further antibiotic therapy. At 08:00 she had back and lower abdominal pain. Her heart rate was 106/minute, blood pressure 86/72 mm Hg and saturation 92% on oxygen at 4 L/minute. Her lips were "dusky". Her abdomen was non-tender. Her supplemental oxygen was increased. Shortly after 08:00 the nurse was unable to locate fetal heart tones. At 08:30 intrauterine fetal death was confirmed by ultrasound examination. When informed of this, the patient was upset. Her abdomen was non-tender. She requested removal of her bladder catheter so she could go to the bathroom "normally". She ambulated to the bathroom, off supplemental oxygen without difficulty, but shortly following this, a nurse was unable to draw blood for blood counts, type and cross-match, and the patient's blood pressure was found to be in the 70s systolic. She was given another 500 ml bolus of normal saline. Over the next 30-45 minutes, the patient had progressive back and lower abdominal pain. She thought she was having uterine contractions. Her abdomen was moderately firm. At 09:00 she was given ceftriaxone (1 gram intravenously). The patient was alert, oriented and talkative, but her skin was becoming cool, and repeated attempts to draw blood and obtain blood pressure by Doppler were unsuccessful.

At 09:25 the patient was transferred to an ICU, where she had dyspnea. She was pale and lethargic, with cold extremities, mottled skin, and fundal tenderness. Bilateral femoral catheters were placed, and aggressive fluid resuscitation started. At 09:30 her temperature was 34.6^o^ C, pulse 106/minute, blood pressure 76 mm Hg systolic and respirations 34/minute. At 10:00, her hemoglobin was 13.4 g/dL, platelets 154,000/mm^3^, INR 0.97, PTT 25.6 seconds and fibrinogen 433 mg/dL. At 10:10 her temperature was 34.5^o^ C, pulse 102/minute, blood pressure 85/46 mm Hg, respiratory rate 36/minute and oxygen saturation 95% on 100% oxygen by non-rebreather mask. She had decreased air entry bilaterally, shallow breathing and pulmonary crackles over the right lower lobe. She developed respiratory distress and her saturation began to drop. The patient was intubated and, shortly following intubation, at 10:25, she became pulseless and cardiopulmonary resuscitation was started. At 10:45 an infusion of dopamine was started, and arterial blood showed pH 7.17, PCO2 59 mm Hg, PO2 25 mm Hg and bicarbonate 20.7 mEq/L. At 10:55 the patient's white blood cell count was 9,600/mm^3^ (neutrophils 80%, lymphocytes 15%, monocytes 3%, bands 2%), hemoglobin 13.6 g/dL, platelets 61,000/mm^3^, INR 1.58, PTT 32.9 seconds and fibrinogen 216 mg/dL. After thirty minutes of resuscitation, the patient's abdomen was tense and fluids would not flow through the femoral lines. The membranes were ruptured, releasing a large amount of bloody fluid.

Cesarean section was performed at 11:00, revealing approximately one liter of clotted blood behind the placenta, and placental abruption was diagnosed. The stillborn fetus was delivered with placenta immediately following. Epinephrine infusion was started at 11:10. The patient continued to have heavy vaginal bleeding, with estimated blood loss of 2 liters. She received 10 units of red blood cells and 4 units of fresh frozen plasma in addition to 5 liters of normal saline. Attempted resuscitation was halted and death pronounced at 11:25.

### Case 3 Autopsy Findings

Autopsy demonstrated numerous small pulmonary blood vessels containing aggregates of platelets, lipid vacuoles, aggregates of neutrophils, occasional cytokeratin-positive cells, rare condensed fibrin emboli, three probable syncytiotrophoblasts (one cytokeratin positive on immunohistochemical stain), and a fragment of calcified material. Both lungs had red, congested and edematous parenchyma. The left lung weighed 515 grams and the right lung 600 grams. Microscopic examination demonstrated moderate acute pneumonia in right middle and lower lobes with abundant fibrin exudate, and foci of very early acute pneumonia in left upper and lower lobes and right upper lobe with abundant fibrin exudate. There were serous pleural effusions, 300 ml left, 500 ml right.

The heart showed acute cor pulmonale with moderate right ventricular dilatation. There was also moderate dilatation of the left atrium and mitral valve prolapse with moderate redundant tissue, particularly of the posterior leaflet. Microscopic examination of mitral valve showed moderate fibrous and myxoid expansion. The liver showed severe passive congestion in the periphery. There were numerous sites of hemorrhage in lungs, heart, thoracic aorta, liver, hypopharynx, duodenum, stomach, left ovary, left renal pelvis and bladder.

Approximately 20% of the placenta was detached from the uterus, supporting the diagnosis of placental abruption. The uterine placental site had an attached fragment of placenta including villi and calcified material. The placenta was thin and small, <10th percentile for gestational age.

## DISCUSSION

Amniotic fluid embolism is a catastrophic, often fatal complication of pregnancy, doubly worrisome for the obstetrician as advocate for both mother and baby.^1^ In typical cases, a patient in labor suffers the sudden onset of hypotension (with systolic blood pressure <90 mmHg or cardiorespiratory arrest), and respiratory compromise (with dyspnea, cyanosis or oxygen saturation <90%), and disseminated intravascular coagulation.^2^ What causes this syndrome? The pathophysiology of amniotic fluid embolism is complicated and incompletely understood. The initial phase seems to be a form of obstructive shock, partly from mechanical obstruction of pulmonary vasculature by amniotic debris and partly from cytokine-mediated pulmonary vasoconstriction.^1^^,^ 3 The next phase seems to be a form of cardiogenic shock.^1^ This is followed by disseminated intravascular coagulation.^1^^,^ 3 Passage of some fetal and placental antigens into the maternal circulation appears to be ubiquitous during delivery, but only a few mothers develop amniotic fluid embolism syndrome from this, so one of the greatest needs in the understanding of the condition is identification of what causes those few mothers to have this catastrophic reaction. Some have placenta previa, 10% of 59 patients with typical amniotic fluid embolism in one recent international registry study.^2^ Placenta previa could conceivably predispose to a large bolus of amniotic fluid entering the maternal circulation from placental rupture during the course of delivery in immunologically susceptible patients. In that recent international registry study, 66% of the 59 women with typical amniotic fluid embolism reported a history of atopy, or allergy to latex, medication or food, compared to 31% of the obstetric population delivered at the authors' hospital.^2^ Some sort of immunological hyperreactivity state could possibly predispose to amniotic fluid embolism syndrome, but no demographic or clinical risk factors are sufficiently studied to justify alterations in standard obstetrical care in anticipation of possible amniotic fluid embolism.^1^^-^ 3

Whatever the pathophysiology of amniotic fluid embolism, prompt therapy can be life-saving. In one case at a community teaching hospital, after 75 minutes of cardiopulmonary resuscitation, a cardiac surgeon was recruited to institute extracorporeal membrane oxygenation; this, together with massive transfusion including 154 units of red blood cells, saved the mother's life. She was doing well, without deficits, at 3-year follow-up.^4^ In a case at an urban teaching hospital, after apparently about 30 minutes of cardiopulmonary resuscitation, transesophageal echocardiography revealed evidence of acute right ventricular failure, the initial phase of the syndrome, which was treated with inotropic agents and inhaled nitric oxide, but within minutes, the patient began having generalized bleeding, which was treated successfully with massive transfusion including 15 units of red blood cells, tranexamic acid and hysterectomy.^1^ Echocardiography is one tool for differentiating amniotic fluid embolism from other conditions that cause sudden cardiopulmonary collapse during labor such as pulmonary thromboembolism, hemorrhage, venous air embolism, anaphylaxis, high cephalad spread of neuraxial anesthetic agent, peripartum cardiomyopathy, acute myocardial infarction and eclampsia.^1^ Coagulation testing may offer other tools for the diagnosis of amniotic fluid embolism. Low fibrinogen, especially in a ratio with blood loss, may occur early in amniotic fluid embolism.^5^ Bedside point-of-care thromboelastography could provide evidence of coagulopathy faster and earlier than blood testing in the laboratory.^6^

Amniotic fluid embolism syndrome is rare and unpredictable, which makes it hard to suspect, but preventing a fatal outcome requires rapid, often drastic therapy. The three cases of our series can provide lessons about when to suspect the diagnosis and need for rapid drastic therapy. In the first case, the patient had a generalized seizure at the onset. Seizures are the classic manifestation of eclampsia, but this can usually be differentiated from amniotic fluid embolism by the lack of hypertension and other features of pre-eclampsia.^1^ Seizures were a feature of amniotic fluid embolism in 21% of the typical cases in the recent international registry study.^2^ The sudden onset of seizure activity in association with oxygen desaturation requiring reintubation 5 minutes after extubation from anesthesia for a cesarean section for placental abruption has been reported as a clue to amniotic fluid embolism.^7^ The sudden onset of seizures in association with hypotension or respiratory compromise can be a clue to the diagnosis of amniotic fluid embolism.

In the second case in our series, the onset of amniotic fluid embolism was heralded by dyspnea, blurred vision, and headache, shortly followed by chest pain and decreased responsiveness. In the recent international registry study, 64% of the 59 patients with typical amniotic fluid embolism had dyspnea, 54% "neurological injury", 13% chest pain and 6% headache, but 100% had hypotension, which our second patient lacked at the onset.^2^ In a recent French series of 42 cases, 73% of the patients had premonitory signs, including 29 with neurological signs (confusion, agitation or sensation of doom), 17 with respiratory signs (not further specified) and 12 with fetal heart rate abnormalities.^8^ It Is difficult to suspect amniotic fluid embolism with such nonspecific early signs and symptoms, but the best chances for saving the mother's and baby's lives are early in the course of the disease. Perhaps the lesson is to remember the possibility of amniotic fluid embolism for the differential diagnosis with the sudden onset of such symptoms and have a low threshold for doing bedside point-of-care ultrasound examination to look for evidence of embolism, pulmonary hypertension or right ventricular overload.

In the third case in our series, the onset of amniotic fluid embolism is very difficult to identify, partly because the patient was admitted with dyspnea due to asthma and pneumonia, and partly because the symptom of dyspnea was followed by back pain, then nausea, then dizziness, then not feeling well, light-headedness and sweatiness, which is a cascade of symptoms not at all suggestive of amniotic fluid embolism. This concatenating cavalcade of symptoms is suggestive of some condition outside the respiratory tract, beyond asthma and pneumonia, especially in association with the onset of hypotension then discovered. Perhaps the lesson from this is one familiar to geriatricians, but not necessarily to obstetricians: Hickam's dictum that a patient may have as many diseases as she darn well pleases.^9^ One could hypothesize that the patient's back pain was from placental abruption and that her nausea was associated with it in a way similar to patients with appendicitis or cholecystitis, who usually have nausea in association with their abdominal pain. The patient then reported dizziness, which may have been what was subsequently termed light-headedness and a manifestation of impaired cerebral circulation as the patient lost one liter of blood into a retroplacental hematoma. The patient had malaise and sweatiness along with the light-headedness, which could be attributed to incipient shock. Placental abruption may have caused amniotic fluid embolism in this complicated case. The detailed presentation of it allows clinician readers to draw additional lessons from this case.

In the first case in our series, the life of the baby was saved with delivery 20 minutes after the onset of the amniotic fluid embolism syndrome. In the second case in our series, the life of the baby was saved with delivery 40 minutes after the onset of the amniotic fluid embolism syndrome. Some would recommend that the baby should be delivered no more than 4 minutes after amniotic fluid embolism is diagnosed.^10^ The condition can occur following an apparently uneventful delivery. In a recently reported case, approximately 30 minutes after a normal vaginal delivery, a 19-year-old patient developed dyspnea with oxygen saturation 37% and hypotension with blood pressure 82/52 mm Hg. She rapidly required intubation and four-agent vasopressor support with norepinephrine, phenylephrine, vasopressin, and epinephrine. Echocardiography and computed tomography showed evidence of amniotic fluid embolism. Supportive therapy with veno-arterial extracorporeal membrane oxygenation was weaned on day 4 and she was discharged from the hospital on day 8.^10^ Rapid diagnosis and drastic therapy can be lifesaving. Our series of three cases is reported as a reminder for experienced clinicians and reported in detail for case-based medical education of clinicians in training.^11^

## CONCLUSION

There is no specific test or treatment for amniotic fluid embolism. Therapy is only supportive, but only supportive therapy is needed because completion of delivery removes the source of the antigens triggering the storm of immunological hyperreaction. The challenge lies in the suddenness and the severity of the syndrome. Therapy must be rapid and often drastic to be successful. The three cases of this report are detailed to help clinician readers maintain amniotic fluid embolism in their differential diagnosis for peripartum events, rapidly recognize this rare syndrome when they encounter it, and institute the therapy needed to save the lives of mother and fetus.
